# High expression of cancer stem cell markers in cholangiolocellular carcinoma

**DOI:** 10.1007/s00595-012-0437-9

**Published:** 2012-11-29

**Authors:** Shuichi Iwahashi, Tohru Utsunomiya, Mitsuo Shimada, Yu Saito, Yuji Morine, Satoru Imura, Tetsuya Ikemoto, Hiroki Mori, Jun Hanaoka, Yoshimi Bando

**Affiliations:** 1 Department of Surgery, Institute of Health Biosciences, The University of Tokushima Graduate School, 3-18-15 Kuramoto-cho, Tokushima, 770-8503 Japan; 2 Department of Molecular and Environmental Pathology, Institute of Health Biosciences, The University of Tokushima Graduate School, 3-18-15 Kuramoto-cho, Tokushima, 770-8503 Japan

**Keywords:** Cholangiolocellular carcinoma, Hepatic stem cells, Combined hepatocellular cholangiocarcinoma, Cancer stem cell marker

## Abstract

**Purpose:**

Cholangiolocellular carcinoma (CLC) is an extremely rare malignant liver tumor. It is thought to originate from the ductules and/or canals of Hering, where hepatic stem cells (HpSC) are located, but there are few reports on cancer stem cell markers in CLC. Thus, we evaluated the significance of cancer stem cell markers, including CD133, CD44, and EpCAM, in CLC.

**Methods:**

The subjects of this study were three patients with CLC and one patient with an intermediate type of combined hepatocellular cholangiocarcinoma (CHC). The cancer cell markers, CK7, CK19, and EMA, were evaluated immunohistochemically.

**Results:**

Histological examination of the CLC revealed morphologically cholangiolar features and immunohistochemical examination revealed positivity for CD133, CD44, and EpCAM. On the other hand, in the intermediate type of CHC, only CD44 was positive, whereas CD133 and EpCAM were negative.

**Conclusion:**

CLC may have stronger features derived from HpSCs than an intermediate type of CHC.

## Introduction

Cholangiolocellular carcinoma (CLC) is a rare primary hepatic tumor. It was first described by Steiner et al. [1] and Steiner and Higginson [2] subsequently reported that it was characterized by small cords resembling cholangioles (canals of Hering) and ductular reaction-like anastomosing glands in abundant fibrous stroma. The canals of Hering are found in portal tracts of all sizes, where they connect with the bile duct. The small cells of the canals of Hering have a basement membrane like the more distal tree, but an apical surface that appears similar to hepatic canalicular membrane. CLC was considered to have “junctional potentialities” because some CLC cases had hepatocellular carcinoma (HCC)-like features. Based on its distinctive histological features, CLC is thought to originate from the ductules and/or canals of Hering where hepatic stem cells (HpSC) are located [2, 3].

A small proportion of tumors composed mostly of combined hepatocellular cholangiocarcinomas (CHC) shows a mixture of hepatocellular and glandular features [4, 5]. A subset of primary liver carcinomas shows features suggesting that they arise from transformed progenitor cells, which have the bipotential to differentiate into both hepatocytes and cholangiocytes. Taguchi et al. [6] classified CHC into three types: type I, in which there are clearly defined areas of HCC and CC; type II, in which the HCC and CC areas are contiguous with an intervening area of transition; and type III, in which the tumor is not readily classifiable as HCC or CC, and is composed of tumor cells showing morphological features intermediate between HCC and CC. Robrechts et al. [7] reported a case of ‘liver tumor of intermediate (hepatocyte-bile duct cell) phenotype’, consisting of small cells with a phenotype intermediate between hepatocytes and cholangiocytes and an intermediate type between HCC and CC, originating from HpSCs [7, 8].

The cancer stem cell (or tumor-initiating cell) concept is that a subset of cancer cells possesses stem cell features indispensable for a tumor. CD133 is expressed in normal and malignant stem cells of the neural, hematopoietic, epithelial, hepatic, and endothelial lineages [9–11], suggesting that CD133 is a common marker to detect normal cells and CSCs. CD44 has also been evaluated as a cancer stem cell marker in solid tumors and in fact, served alone as a cancer stem cell marker in head and neck carcinoma [12]. Recent studies have also indicated that epithelial cell adhesion molecules (EpCAM) are a biomarker for HpSCs because they are expressed in HpSCs and hepatoblasts [13, 14]. Yamashita et al. [15] reported that both EpCAM and CD133 may be hepatic cancer stem cell markers specifically in HpSC-HCC; however, very few reports have investigated the cancer stem cell marker in CLC. Thus, we evaluated the significance of cancer stem cell markers in CLC versus an intermediate type of CHC.

## Materials and methods

### Patients

The subjects of this study were three patients with CLC and one patient with intermediate type CHC, who underwent surgical treatment between 1995 and 2009 at the University of Tokushima. Surgical specimens were examined pathologically using hematoxylin and eosin-stained tissue preparations. This study was authorized in advance by the Institutional Review Board of the University of Tokushima Graduate School of Medicine, and all the patients provided written informed consent.

### Immunohistochemical staining

Formalin-fixed, paraffin-embedded samples were used in the study. Sections were serially cut at 5 μm, then dewaxed, deparaffinized in xylene, and rehydrated through a series of graded alcohols. For better antigen retrieval, the samples were boiled for 20 min in a microwave oven in a citrate buffer (pH 6.0). Endogenous peroxidases were blocked by 0.3 % hydrogen peroxidase treatment for 30 min. The samples were incubated in 5 % goat serum for 60 min to prevent nonspecific antigen binding. The slides were incubated with primary antibodies overnight at 4 °C. We used the following primary antibodies and dilutions: 1:100 dilution of a mouse monoclonal antibody for CD133 (Abcam), 1:100 dilution of a rabbit monoclonal antibody for CD44 (Abcam), 1:50 dilution of a mouse monoclonal antibody for EpCAM (Santa Cruz Biotechnology), 1:200 dilution of a mouse monoclonal antibody for alpha-fetoprotein (AFP) (Abcam), and 1:50 dilution of a mouse monoclonal antibody for forkhead box protein A2 (FoxA2) (Abcam). The secondary peroxidase-labeled polymer conjugated to goat anti-mouse immunoglobulins was applied for 60 min. The sections were developed in 3,3-diaminobenzidine and counterstained with Mayer’s hematoxylin. The slides were dehydrated through graded alcohols and coverslips were applied. For unbiased immunohistochemical staining, two investigators decided independently on the positive cells on each slide. CD133, CD44, and EpCAM positivity was recorded if any cells were stained in the cytoplasm of the tumor [16]. The frequency of positive cells in the tumor was as low as 0.5–2.0 %, consistent with previous reports [16, 17].

### Immunofluorescent staining

To confirm both CD44 and EpCAM expression in the CLC cancer cells, immunofluorescent staining was performed on sections of the CLC specimens. For two-color immunofluorescent staining, the primary antibody for CD44 was detected with Alexa Fluor 488-conjugated gout anti-rabbit IgG (Invitrogen) (1:2000 dilution). The other primary antibody for EpCAM was detected with Alexa Fluor 594-conjugated gout anti-mouse IgG (Invitrogen) (1:2000 dilution) for 60 min. Finally, the slides were washed in 0.1 % Triton X-100 in PBS, then viewed and photographed under a confocal laser scanning microscope (Leica Microsystems, Wetzlar, Germany).

## Results

Table 1 summarizes the backgrounds of the four patients. In CLC, AFP was positive/negative (1/2), whereas vitamin K absence-II (PIVKA-II) and CEA (carcinoembryonic antigen) were negative, and CA19-9 (carcinoma 19-9) was positive/negative (1/2). In CHC, AFP, PIVKA-II, and CA19-9 were positive, whereas CEA was negative.

**Table 1:** Background in the patients with cholangiolocellular carcinomas and combined hepatocellular cholangiocarcinoma

	CLC			CHC
	Case 1	Case 2	Case 3	
Age/gender	61/M	72/F	71/M	67/M
Hepatitis virus	HBV (+)	HCV (+)	nonBnonC	HCV (+)
AFP (ng/ml)	67	6	6	8287
AFP-L3 (%)	80	(−)	(−)	NA
PIVKA-II (mAU/ml)	19	26	10	6
CEA (ng/ml)	1.1	0.8	1.4	1.2
CA19-9 (U/ml)	13	187	9	222
DUPAN-II (U/ml)	<25	<25	NA	NA
Span-I (U/ml)	34	33.7	NA	NA

*CLC* cholangiolocellular carcinoma, *CHC* combined hepatocellular cholangiocarcinoma, *HBV* hepatitis B virus, *HCV* hepatitis C virus, *AFP* alpha-fetoprotein, *PIVKA-II* vitamin K absence-II, *CEA* carcinoembryonic antigen, *CA19-9* carcinoma 19-9, *DUPAN-II* duke pancreatic monoclonal antigen type II, *Span-I* s-pancreas-1 antigen

**Fig. 1:**
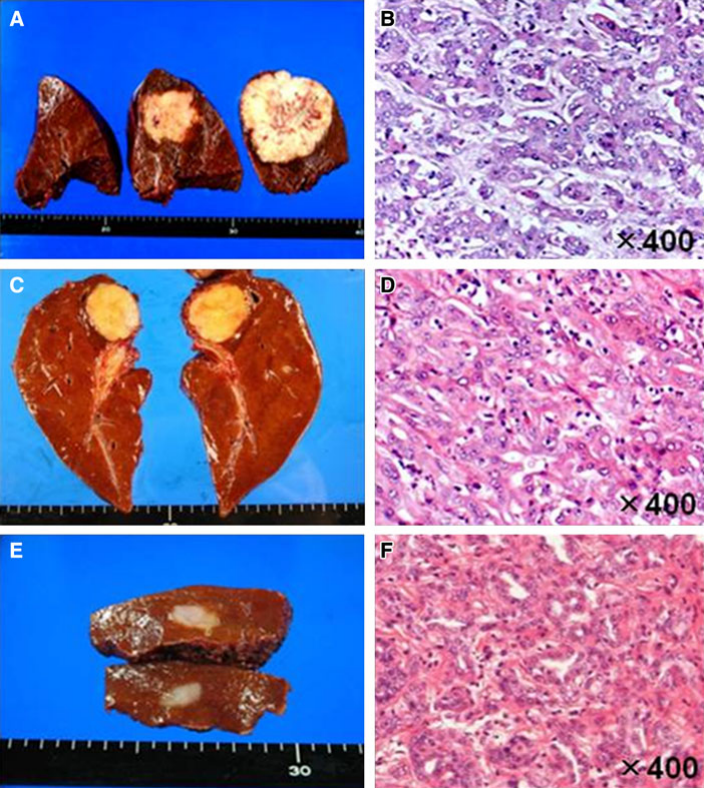
Resected specimens from case 1 (**a**), case 2 (**c**), and case 3 (**e**). Histological findings of the specimens from case 1 (**b**), case 2 (**d**), and case 3 (**f**) (H&E stains)

In CLC case 1, the resected tumor was whitish and measured 80 × 75 mm. Histological examination revealed a tumor invasively proliferated in a duct-like configuration without the production of mucinous fluid. Small ductules showed an anastomosing pattern, and some of the tumor cells proliferated by replacing the surrounding normal hepatocytes. In CLC case 2, the tumor was slightly xanthochromatic and measured 40 × 45 mm. The histological findings were similar to those of CLC case 1. In CLC case 3, the tumor measured 10 × 15 mm and its histological findings were similar to those of CLC cases 1 and 2 (Fig. 1). In the case of intermediate type CHC, the tumor was arranged in strands with vague gland-like structures.

**Fig. 2:**
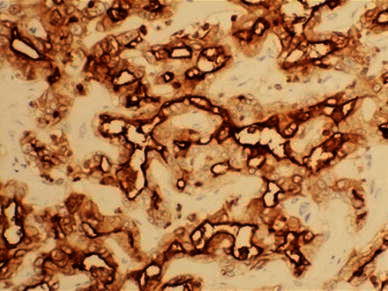
EMA was strongly positive in the cholangiolocellular carcinoma (CLC), mainly localized on the apical surface of the tubules

**Fig. 3:**
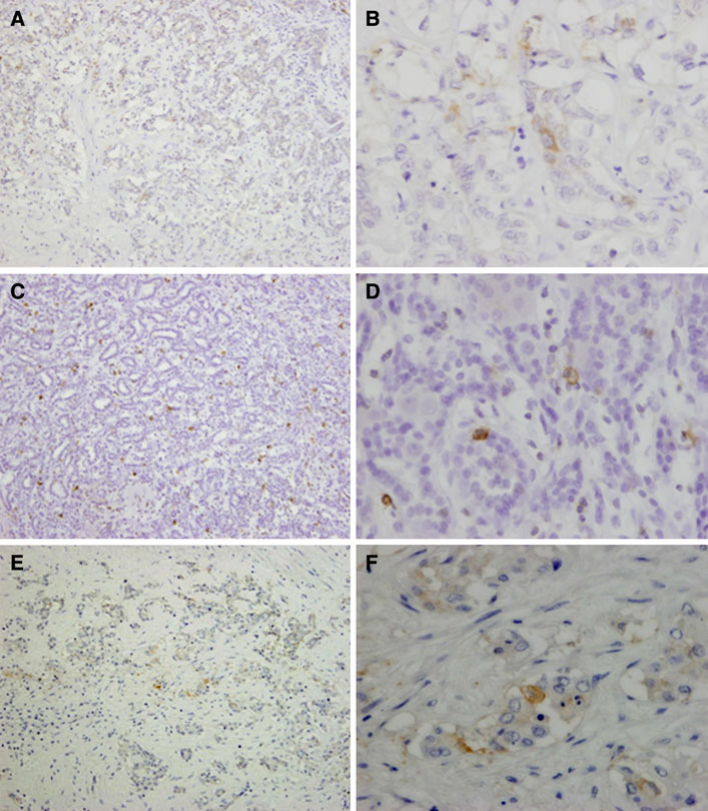
Immunohistochemical expression of the cancer stem cell markers [CD133 ×100 (**a**), ×400 (**b**), CD44 ×100 (**c**), ×400 (**d**), EpCAM ×100 (**e**), ×400 (**f**)] in the cancer cells

**Fig. 4:**
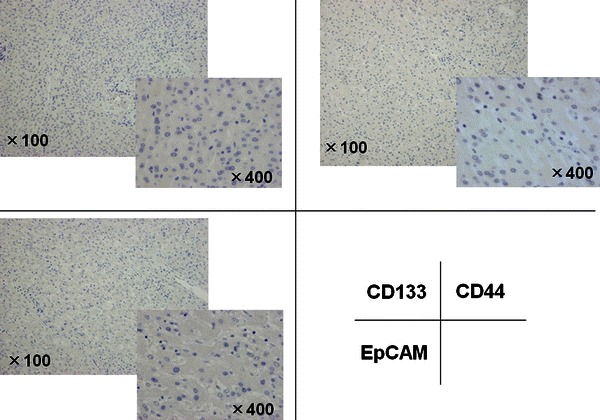
Immunohistochemical expression of the cancer stem cell markers (CD133, CD44 and EpCAM) in the non-cancer cells

**Fig. 5:**
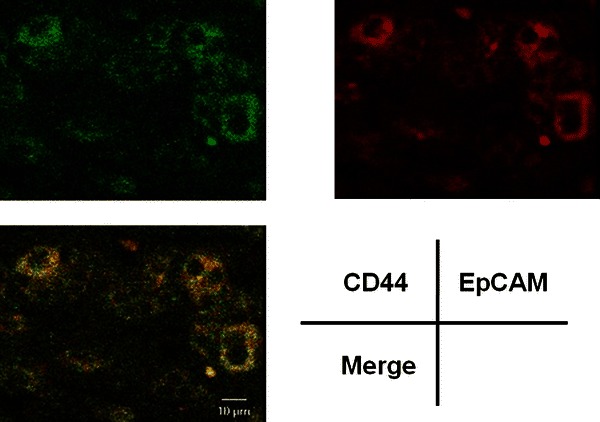
Two-color immunofluorescent staining for CD44 and EpCAM in the cholangiolocellular carcinoma cells

**Fig. 6:**
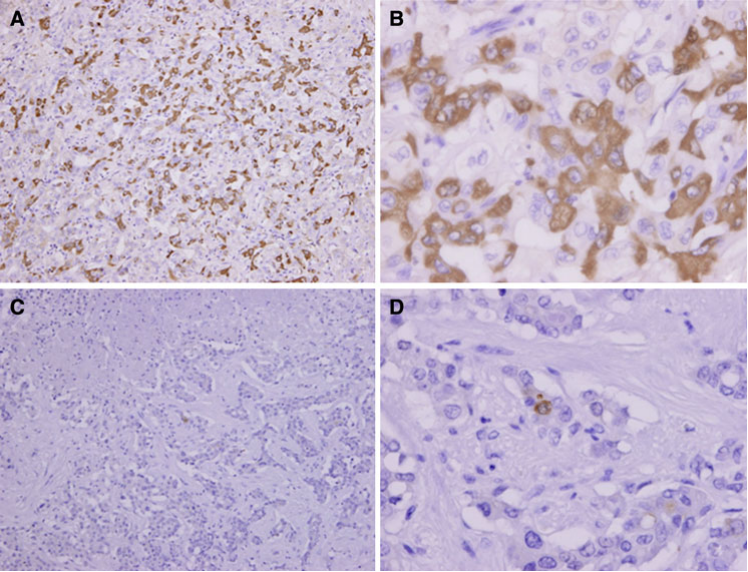
Immunohistochemical staining of HpSC markers [AFP ×100 (**a**), ×400 (**b**), FoxA2 ×100 (**c**), ×400 (**d**)]

Immunohistochemical analysis revealed positivity for cytokeratin (CK) 7 and CK19 in all four patients. Epithelial membrane antigen (EMA) was strongly positive in CLC and slightly positive in CHC. In EMA, the stain was strongly positive and found to be localized on an apical surface of the tubules (Fig. 2). Immunostaining of the cancer stem cell markers in tumor cells revealed positivity for CD133, CD44, and EpCAM in the CLC. On the other hand, in the intermediate type of CHC, only CD44 was positive, whereas CD133 and EpCAM were negative (Fig. 3; Table 2) and no positive cells were found in the non-tumorous cells (Fig. 4). Two-color immunofluorescent staining for CD44 and EpCAM confirmed both cancer stem cell markers co-expressed in the serial section of CLC (Fig. 5). In addition, we investigated the AFP and FoxA2 expressions as HpSC markers [18, 19] and confirmed positive staining for AFP and FoxA2 in CLC (Fig. 6).

**Table 2:** Immunohistochemical staining in cholangiolocellular carcinomas and combined hepatocellular cholangiocarcinoma

	CD133	CD44	EpCAM	CK7	CK19	EMA^a^
CLC						
Case 1	(+)	(+)	(+)	(+)	(+)	(++)
Case 2	(+)	(+)	(+)	(+)	(+)	(++)
Case 3	(+)	(+)	(+)	(+)	(+)	(++)
CHC	(−)	(+)	(−)	(+)	(+)	(+)

*CLC* cholangiolocellular carcinoma, *CHC* combined hepatocellular cholangiocarcinoma, *EpCAM* epithelial cell adhesion molecule, *CK7* cytokeratin 7, *CK19* cytokeratin 19, *EMA* epithelial membrane antigen

^a^In EMA, strongly positive (++) was that the stain showed the localization in apical surface of the tubules

## Discussion

CLC is an extremely rare primary liver tumor with a frequency as low as 0.56 % in Japan [20]. The clinicopathological features of CLC have not been fully clarified because of its rarity, although Komuta et al. [21] did recently report on the clinicopathological characteristics of 30 patients with CLC. They found that CLC was composed of a mixture of small monotonous glands and antler-like anastomosing patterns, with an abundant hyalinized and/or edematous fibrous stroma with lymphocytic infiltration. The tumor cells were cuboidal and smaller than normal hepatocytes, with scanty eosinophilic cytoplasm, round nuclei, and distinct nucleoli. Both the HCC-like trabecular area and the CC area were composed of less than 10 % of the tumor. CLC, HCC-like trabecular, and CC areas coexisted in varying degrees in the tumors and these three different histological patterns showed transitions between each other [21].

CLC is thought to originate from the ductules and/or canals of Hering, where HpSCs are located [2, 3]. Recent studies confirm that HpSCs do exist in the smallest and most peripheral branches of the biliary tree, the ductules, and the canals of Hering [22]. HpSCs have long been recognized to play important roles in liver regeneration and hepatocarcinogenesis [23–25]. Therefore, HpSC can differentiate toward both hepatocytes and cholangiocytes and can give rise to HCC/CC on its way to differentiation. When HpSCs develop into cancer, the tumors have a spectrum of phenotypes with varying hepatocellular and cholangiocellular differentiation characteristics [26–28].

Recent studies have shown that EpCAM is a biomarker for HpSCs [9, 13, 14] and that EpCAM-positive HCC cells have stem cell features [15], indicating that EpCAM is one of the cancer stem cell markers of HCC [29]. CD133 and CD44 have also been recognized as HpSCs biomarkers [30, 31] and CD133- and CD44-positive cells represented cancer stem cells in HCC [32]. All these reports exemplify the strong association of HpSCs with cancer stem cells. Intriguingly, a recent study demonstrated that a protein, cullin-3, prevented HpSCs from becoming tumor-initiating cells [33]. Further investigations are warranted.

Komuta et al. [21] found that the CD133 mRNA level was significantly higher in CLC than that in HCC and they also demonstrated higher expression of CD133 in the HpSCs/ductular reaction than in nontumoral hepatocytes. We also performed immunostaining of AFP and FoxA2 as HpSC markers [18, 19] and confirmed positive staining of these markers in CLC. Based on these findings, they suggested that HpSC might be the origin of CLC. The present study revealed that CD133, CD44, and EpCAM were all positive in CLC, whereas only CD44 was positive and CD133 or EpCAM were negative in the intermediate type of CHC. These findings suggest that CLC may have features more strongly derived from the hepatic progenitor cells than the intermediate type of CHC.
